# Extending the toolbox for RNA biology with SegModTeX: A polymerase-driven method for site-specific and segmental labeling of RNA

**DOI:** 10.21203/rs.3.rs-2782805/v1

**Published:** 2023-04-06

**Authors:** Raphael Haslecker, Vincent V. Pham, David Glänzer, Christoph Kreutz, Theodore Kwaku Dayie, Victoria M. D’Souza

**Affiliations:** 1 Department of Molecular and Cellular Biology, Harvard University, Cambridge, MA, 02138, USA.; 2 Institute of Organic Chemistry and Center for Molecular Biosciences Innsbruck (CMBI), University of Innsbruck, Innrain 80/82, 6020 Innsbruck, Austria.; 3 Department of Chemistry and Biochemistry, University of Maryland, College Park, MD 20782, USA

## Abstract

RNA performs a wide range of functions regulated by its structure, dynamics, and often post-transcriptional modifications. While NMR is the leading method for understanding RNA structure and dynamics, it is currently limited by the inability to reduce spectral crowding by efficient segmental labeling. Furthermore, because of the challenging nature of RNA chemistry, the tools being developed to introduce site-specific modifications are increasingly complex and laborious. Here we use a previously designed *Tgo* DNA polymerase mutant to present SegModTeX — a versatile, one-pot, copy-and-paste approach to address these challenges. By precise, stepwise construction of a diverse set of RNA molecules, we demonstrate the technique to be superior to RNA polymerase driven and ligation methods owing to its substantially high yield, fidelity, and selectivity. We also show the technique to be useful for incorporating fluorescent- and a wide range of other probes, which significantly extends the toolbox of RNA biology in general.

## Introduction

RNA is positioned at a critical cross-section of biology: it not only disseminates genetic information but also folds into structures to mediate many biological functions. This is due to its capacity to form numerous basepair-types beyond the canonical Watson-Crick pairs, often providing the ability to sample multiple conformations that are critical to drive function^1–3^. Additionally, more and more nucleotide modifications at predefined sites are known to further expand the structural, mechanistic, and functional repertoire of RNA^4^. It is therefore important to understand RNA structures in their various conformations and in the correct chemical context to gain complete insights into its function and the biology it governs.

However, current RNA structural characterization methods suffer from three major drawbacks. The first is specific to NMR; while it is strongly positioned to provide structural data across the full folding landscape of a molecule, it is not conducive for working on large RNAs due to signal overlap and broad linewidths. Second, it is challenging to construct functionally relevant chemical states of RNAs; for example, modifications like N^6^-methyl-adenosine (m^6^A), 5-methyl-cytosine (m^5^C), pseudouridine (Ψ), etc., are difficult to add site-specifically using an enzymatic approach. Third, many biochemical techniques, such as FRET, have limited options on where the probe can be placed and hence are mostly confined to the termini, thus restricting complete characterization of the molecule.^5^

One way to overcome both the size limitations of NMR and site-specific incorporation of modified nucleotides is to construct RNA segments sequentially, each of which can be manipulated for selective incorporation of isotopes (termed segmental labeling) and/or modified rNTPs and analogues^6^. There are three methods currently in use for segmental and site-specific labeling. First, individually synthetized RNA fragments can be ligated with T4 DNA or RNA ligase, which has been successfully used in several studies including an NMR study of 100 kDa HCV IRES RNA^7,8^. It has recently been extended to allow for site-specific addition of single bases at the ligation site using chemical or T7 RNA polymerase synthesis.^9,10^ However, ligation itself has shortcomings such as low yields in individual ligation and preparatory steps, sequence constraints at and around the ligation site, and minimum segment lengths, all of which severely limit its use^7^. Second, position-selective labeling of RNA (PLOR) uses T7 polymerase to extend RNA stepwise by using different unlabeled, labeled, or modified rNTP pools in individual steps^11^. T7 polymerase starts transcription *de novo* and cannot reengage with an RNA:DNA duplex. PLOR tries to circumvent this by keeping the RNA polymerase engaged with the duplex while the rNTP pools are washed on and off^12,13^. However, due to its technical complexity, use of saturating amounts of labeled rNTP pools per wash/step, and limited yield, it has not found widespread use.

Finally, a third approach involves chemical RNA synthesis using phosphoramidite nucleotide analogues. This method easily allows for selective incorporation of modified or isotopically labeled nucleotides^14^. However, chemical synthesis has a length constraint of ~70 nt for NMR spectroscopy. This limitation is imposed by the current step-wise coupling efficiency of ~98% in each step, which leads to overall low yields further diminished by side products during the deprotection steps^15,16^. Furthermore, many phosphoramidite versions of modified rNTPs are not commercially available and thus off limits to most labs^17^. Together, these methods have found limited application in overcoming the size and crowding limitations of NMR as only ~2% of deposited NMR structures in the RCSB database are greater than 70 nucleotides (April 2023) with the largest being 155 nt^8^. Out of these, almost half required the use of segmental labeling via ligation to unambiguously assign resonances. Additionally, all deposited structures that incorporate site-specific RNA modifications either used short, chemically synthesized RNAs or RNAs extracted from biological material, both of which severely limits the type and composition of RNAs that can be studied.

**Figure 1:**
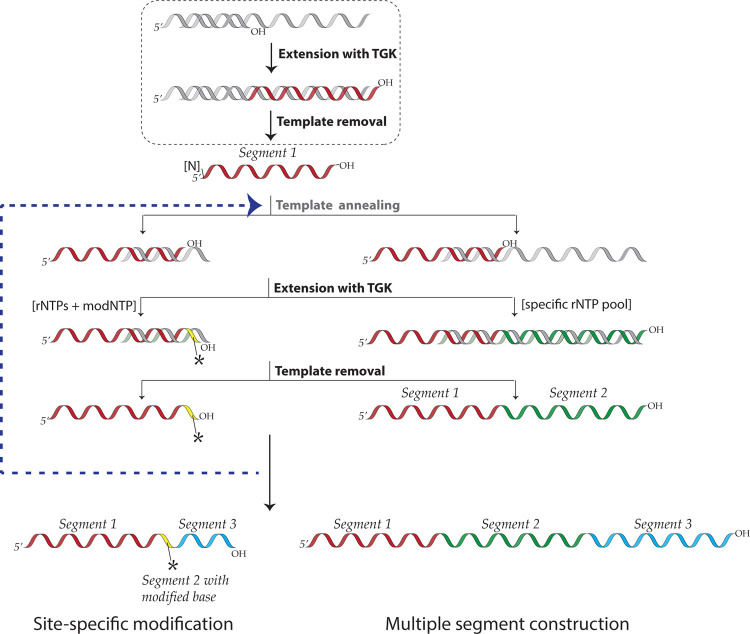
Schematic of the SegModTeX protocol The RNA segment 1 (red) is annealed to the reverse complementary template 1 (grey) encoding the second segment and a region complementary to the 3′-end of segment 1 of ~20nt. Upon extension of segments 2 (yellow and green) using a pool containing the desired modified (left) or isotopically labeled (right) rNTPs, the template is removed and the extended RNA of segment 1+2 can undergo another round of extension (segment 3, blue).

However, while segmental incorporation of nucleotides in RNA is problematic, this is not the case for DNA, and can be easily achieved by DNA primer extension with labeled or modified dNTP pools. This is because DNA polymerases engage an existing primer template duplex and extend from the 3′-end of the primer, allowing the newly synthesized segment to be different from the primer depending on the dNTP mix provided^18^. On the other hand, virtually all RNA polymerases initiate transcription *de novo* and are not thermostable, making segmental additions or melting steps impossible. Nevertheless, Cozens et al. reported a mutant DNA polymerase (TGK) from *Thermophilus gorgonarius (Tgo)* with a unique capability to not only efficiently incorporate rNTPs, but importantly, also extend an RNA primer that is annealed to a DNA template^19^. Building on that work, we characterized and optimized the enzyme’s fidelity, accuracy, versatility, and yield for extending RNA primers/segments. Here we present a method for ‘Segmental labeling and site-specific Modifications by Template-directed eXtension’ (SegModTeX, Fig. 1). We mainly use NMR as proof-of-principle, both to advance the field and due to its ease of detecting modifications and assessing sample quality. Furthermore, we describe a range of NTP analogues that are accepted by the polymerase, thus greatly expanding the toolbox for RNA biology.

## Results

### Extending and modifying RNAs with high efficiency and fidelity by SegModTeX

For high quality and easy purification of TGK polymerase we used an N-terminal GST-tag followed by a PreScission protease cleavage site upstream of the polymerase sequence. We obtained yields of 25 mg of protein per liter of *E. coli* culture which is comparable to that obtained for T7 polymerase^20^.

**Figure 2:**
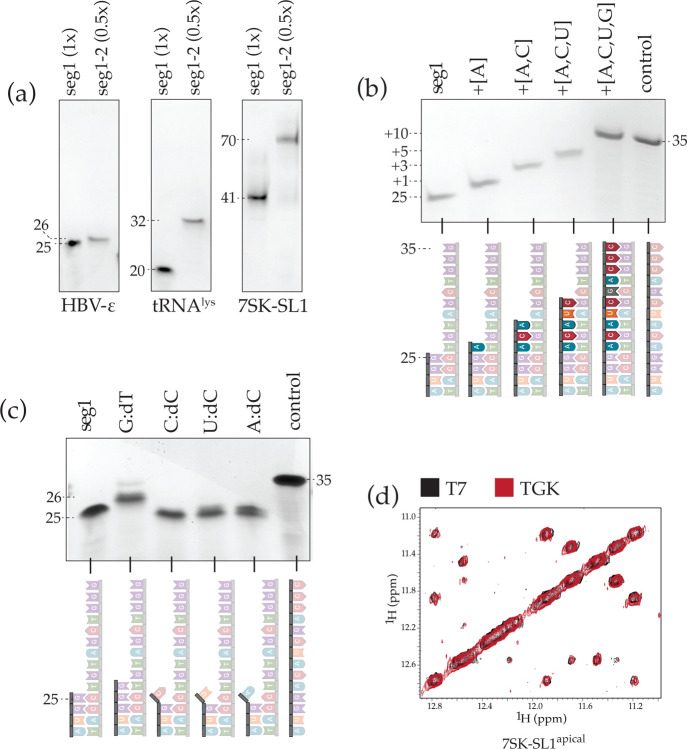
Yield, fidelity, and NMR sample quality of SegModTeX **(a)** RNA sequences from 25 nt HBV-ε seg1 (left), 20 nt tRNA^lys3^ seg1 (center), and 41 nt 7SK-SL1 seg1 (right) extended quantitatively by 1, 12, and 29 nt respectively. Seg1 input equivalent denoted in brackets for SegModTeX products. For larger ssDNA templates full equivalents were not loaded due to high dilution volumes in the DNase treatment. Lack of input segment in product lanes shows complete product turnover. **(b)** 14% polyacrylamide-25% formamide gel showing the accurate incremental extensions of HBV-ε seg1 (25nt, lane 1) based on the combination of NTP mix provided (lanes 2 to 5). SegModTeX reactions proceed only up to the length expected and have a hard-stop when the complementary NTP is missing, highlighting high fidelity, a feature that is a prerequisite for segmental labeling. **(c)** Overlay of two 2D-NOESY NMR spectra of 56 nt 7SK-SL1^apical^ constructed by T7 polymerase (*de novo* synthesis) in black and by SegModTeX: extension of seg1 (28 nt) to seg1+2 (56 nt) in red.

We tested the purified TGK polymerase for properties that would make it conducive for segmental extension and labeling. First, we confirmed the enzyme’s gain-of-function for incorporating rNTPs by extending a diverse set of RNA segments of varying length on DNA templates also of varying lengths. Segment 1 (seg1) of the various lengths and compositions were either made by T7 polymerase or by chemical synthesis, including HBV-epsilon (HBV-ε) (25_seg1_), tRNA^lys3^ (20_seg1_), and 7SK snRNA (41_seg1_). Each seg1 was annealed to a ssDNA template with a complementary 3′-end (~20nt, T_m_: ~65°C) and encoding the sequence of seg2 at the 5′-end. TGK was then used to extend the various seg1 to produce seg1-seg2: HBV-ε (+1 to 26_seg1-seg2_), tRNA^lys3^ (+12 to 32_seg1-seg2_), and 7SK snRNA (+29 to 70_seg1-seg2_). We rigorously tested all relevant parameters, including concentration of template, segments, rNTPs, enzyme, and divalent ions and additionally optimized buffer, pH, annealing conditions, temperature, and time for extension. Unlike T7 polymerase, all SegModTeX reactions were robust and went to completion under the same optimized reaction conditions of 0.1 mM template:seg1, 1.5 mM rNTPs, 0.1 mg/ml TGK, 15 mM MgSO4, 1x ThermoPol buffer (pH: 7.1), at 72 °C for < 90 min. The turnover of the various seg1 to seg1-seg2 appears to be 100% as evidenced by the complete absence of seg1 in the enzymatically extended lanes (Fig. 2a).

Next, as TGK can use an expanded range of rNTP analogues as substrates, we tested if misincorporation of complementary bases occurs, which could lead to increased heterogeneity of the RNA constructs and render the method inadequate for segmental labeling. Therefore, we extended HBV-ε seg1 (25_seg1_) using different combinations of rNTPs (A, AC, ACU, and ACUG) and checked if the enzyme stalls at the predicted site (+1, +3, +5, +10, respectively), or if the polymerase extends beyond that by using the wrong rNTPs. Remarkably, the predicted extensions occurred exactly as delimited by the complementary rNTPs, indicating a low promiscuity of the polymerase (Fig. 2b).

Furthermore, for segmental labeling, it is essential that accurate segment ends are used. In fact, in canonical ligation methods, terminal ribozymes are added to the desired sequence because T7 polymerase has a propensity to add non-templated bases at the end^21,22,23^. This is of special concern with long RNA segments where purification at single-nucleotide resolution is not feasible. Thus, we tested whether TGK retains its DNA polymerase property to discriminate and not use mismatched base pairs as substrates, which would allow for production of only accurate segment junctions^24^. We extended HBV-ε seg1 (25_seg1_) with different versions of mismatched 3′-end base pairs (G:dT, C:dC, U:dC, A:dC) and performed extension assays. Mismatched segments are not extended (Fig. 2c); in fact, even the G:dT pair, which has been shown to be efficiently extended by Taq polymerase shows almost no detectable extension^24^.

Finally, to confirm the high yield and fidelity of SegModTeX, we compared NMR spectra of a 56 nt 7SK-SL1^apical^ RNA made by T7 polymerase to that made by TGK (28nt_seg1_ to 56nt_seg1-seg2_)^25^. 7SK-SL1^apical^ is especially enriched with bulges and non-Watson base pairs which are readily discernable by NMR. Our comparative analysis (Fig. 2d) shows that the sample made by segmental labeling is indistinguishable from that made by T7, emphasizing the high fidelity and accuracy of SegModTeX.

### SegModTeX allows for rapid NMR assignments of large multidomain RNAs

**Figure 3:**
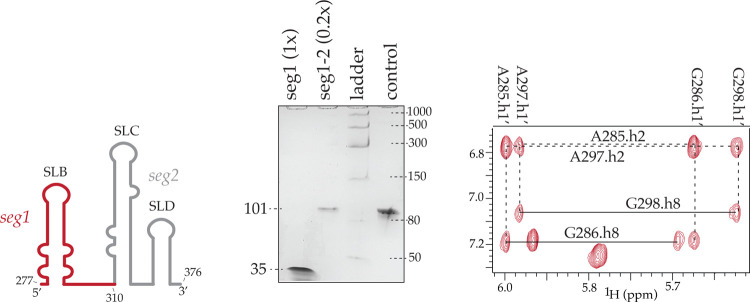
Labeling large RNA segments for crowded spectra (MLV) **Left:** Schematic of the segmental labeling of MLV-BCD. Seg1 (red) is fully protonated, whereas seg2 (grey) is fully deuterated. Center: 10% polyacrylamide-formamide gel showing the extension of RNA seg1 (lane 1) to seg1+2 (lane 2). Seg1 input equivalent denoted in brackets for SegModTeX products. For larger ssDNA templates full equivalents were not loaded due to high dilution volumes in the DNase treatment. Lack of input segment in product lanes shows complete product turnover. **Right:** 2D-NOESY NMR spectra of segmental deuterated MLV-BCD RNA. The vastly simplified spectra allow for easy peak assignment in the context of the full RNA.

Since current segmental labeling techniques are inefficient and laborious, RNA assignments by NMR largely rely on atom- or nucleotide-specific labeling. Atom-specific labeling strategies only allow for assignments of up to 70 nt with ease. Furthermore, deuteration, wherein proton positions are substituted for deuterons, leads to loss of structural information at those sites, which is especially critical for solving the structures of loops and bulges. Nucleotide-specific labeling strategies have been used to investigate RNAs over 100 nt but are a resource- and time-consuming process. It requires the parallel assignments from numerous nucleotide specifically labeled samples; for example, assignments of the MLV-Ψ packaging signal (SL-BCD) required four specifically deuterated samples, four ^15^N/^13^C labeled samples, each of which required 3D and 4D datasets^26^. For comparative analysis, we used SegModTeX to make the three-domain SL-BCD construct wherein the B domain (seg1, 35nt) was left protonated while domains C and D were extended using deuterated rNTPs, rendering these domains invisible to NMR (seg1-seg2, 101nt) (Fig. 3). Thus, use of such segmental labeling would only require three domain-specific protonated samples, each of which can be used to fully assign the respective domain, thus significantly reducing the time required to solve the structure. Furthermore, this strategy can be combined with atom-specific deuteration at ribose moieties to significantly reduce the overlap, and further aid in assignments of full domains on large RNAs^27^.

### SegModTeX allows for multiple extensions without segment length- or sequence constraints

**Figure 4:**
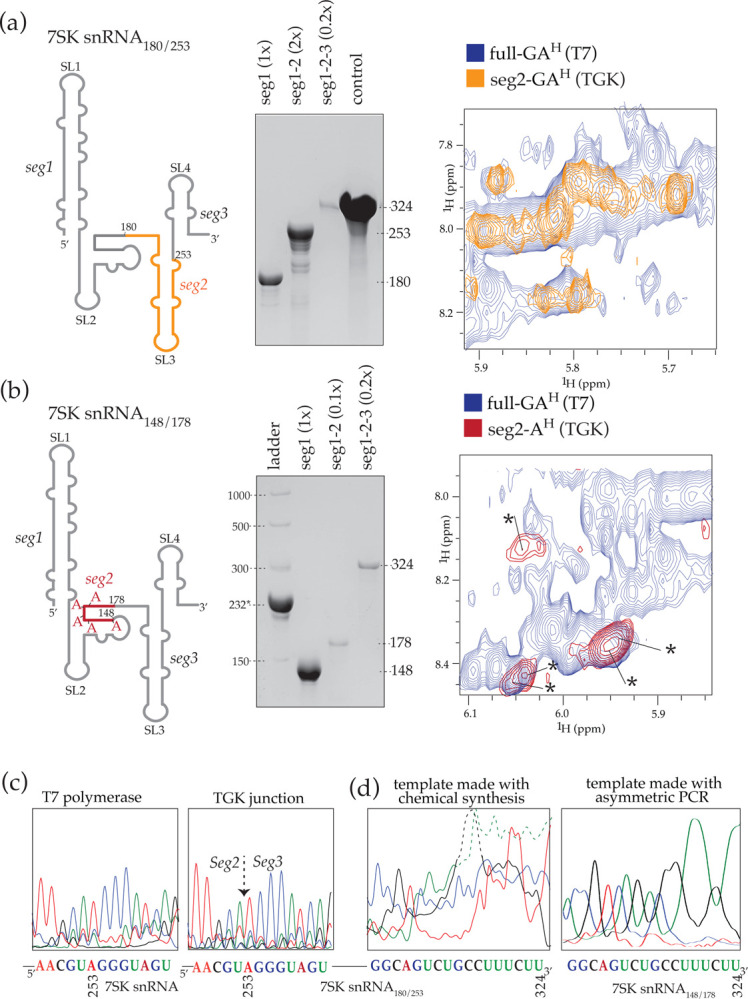
Multi-segment labeling of large 7SK snRNAs **(a) Left:** schematic of the segmental labeling of 7SK snRNA. Seg1 and 3 (grey) are fully deuterated, whereas in seg2 (orange) only C and U are fully deuterated, while all G and A are protonated. **Center:** 6% polyacrylamide-formamide gel showing the stepwise extensions of RNA segments 1 to 3 (lane 1–3). Seg1 input equivalent denoted in brackets for SegModTeX products. **Right:** Overlay of 2D-NOESY NMR spectra of ordinary T7 transcribed C and U deuterated 7SK snRNA (black) and the vastly simplified segmentally labeled 7SK snRNA (orange), where individual cross-peaks are discernable. **(b) Left:** schematic of the segmental labeling of 7SK snRNA. Seg1 and 3 (grey) are fully deuterated, whereas in seg2 (red) G, C and U are fully deuterated, while only the 5 As are protonated. **Center:** 6% polyacrylamide-formamide gel showing the stepwise extensions of RNA segments 1 to 3 (lane 2–4). **Right:** Overlay of 2D-NOESY NMR spectra of ordinary T7 transcribed C and U deuterated 7SK snRNA (black) and the vastly simplified segmentally labeled 7SK snRNA (red), where the individual cross-peaks are discernable. Seg1 input equivalent denoted in brackets for SegModTeX products. For larger ssDNA templates full equivalents were not loaded due to high dilution volumes in the DNase treatment. Lack of input segment in product lanes shows complete product turnover. **(c)** RT-seq of the 7SK snRNA NMR samples at the junction of seg2 and seg3. Sequencing results for a complete T7 polymerase product (left) and a SegModTeX product using ssDNA (right) match. **(d)** RT-seq of the 7SK snRNA NMR samples at the 3′-end. SegModTeX product using chemically synthesized ssDNA template (left) has degenerate ends whereas the product using asymmetric PCR derived template (right) has correct ends.

Assignments of domains in the middle of RNAs, for example domain C in the SL-BCD above would traditionally require two or more steps of differentially labeled segments. Thus, we tested if multiple segments of RNA can be efficiently added using SegModTeX. For this we used two variations of segment designs to produce a 324nt 7SK snRNA. In the first, we designed a multi-step extension protocol to only visualize guanosines and adenosines from residues 181 to 253, while completely deuterating the segments on either end (7SK snRNA_180/253_) (Fig. 4a). In the second, we wanted to visualize only the five adenosines present in regions between 149 and 178, while completely deuterating all other bases (7SK snRNA_148/178_) (Fig. 4b). After extending seg1 to seg1-seg2 and subsequent DNase digestion we repeated SegModTeX using new ssDNA templates to add seg3. Comparison with a fully protonated, full-length 7SK snRNA made by T7 polymerase shows the individually identifiable proton shifts of the specified nucleotides in both samples, for example, five expected adenosines are visible in 7SK snRNA_148/178_ that align well with the resonances of the control sample. Such drastic reduction of spectral complexity should easily allow for structural characterizations of specific domains and/or nucleotides in large RNAs (Fig. 4a, 4b).

To confirm that non-complementary base pairs are not extended (see Fig. 2c) we allowed for incomplete extensions of seq1-seg2 during the extension of 7SK snRNA_180/253_ (Fig. 4a). While shorter fragments would naturally lack complementarity to the next template and thus be excluded from further extensions, mismatched fragments that still anneal to the template would cause inaccurate junctions. Sequencing of 7SK snRNA_180/253_ shows that the junction made in the 7SK snRNA_180/253_ sample is comparable to that region made by non-segmented T7 polymerase transcription, confirming the above observation that SegModTeX does not extend inaccurate or mismatched ends (Fig. 4c). On the other hand, we find that the 3′-end of the 7SK snRNA_180/253_ seg3 has significant heterogeneity. Given the high fidelity of the enzyme, we rationalized this to be the result of the heterogeneity of template sizes produced by chemical DNA synthesis. To circumvent this heterogeneity problem, we tested the use of asymmetric PCR to construct a homogenous ssDNA template. We synthesized the whole 324nt 7SK snRNA template sequence, which was used for the 7SK snRNA_148/178_ synthesis above^28^. As expected, sequencing results showed that the 3′ end was accurately extended, highlighting the ability of SegModTeX to construct RNA segments of any length.

**Figure 5:**
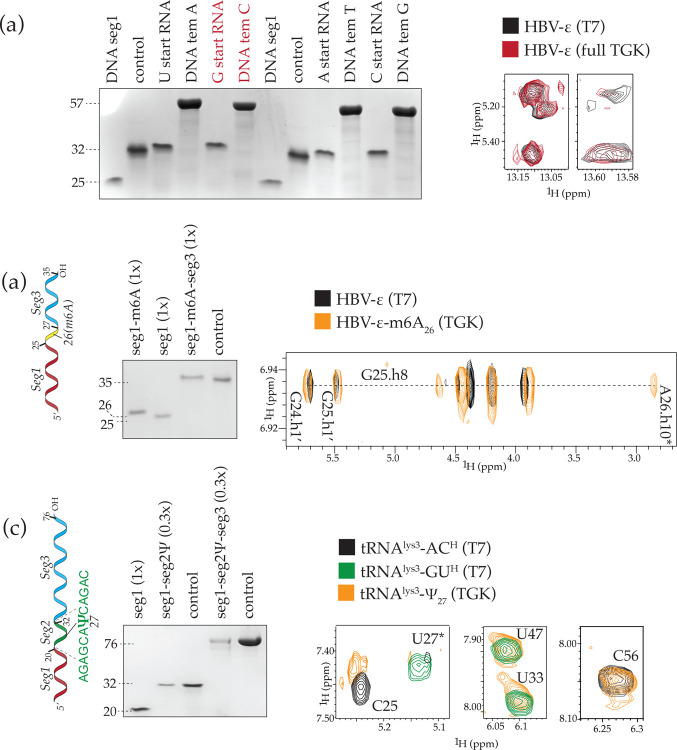
Multi-segment extension with SegModTeX **(a) Left:** DNA primed RNA synthesis via SegModTex. 25 nt DNA seg1 (lane 1) is extended using rNTPs and a 57 nt ssDNA templates (lanes 4, 6, 10, 12) starting RNA synthesis with U, G (red), A, and C, respectively. Upon DNase digestion 32 nt RNA remains (lanes 3, 5, 9, 11). **Right:** Overlay of 2D-NOESY NMR spectra of DNA primed SegModTeX derived HBV-ε-stem1 (red) and the same RNA from T7 transcription (black) **(b) Left:** Schematic and polyacrylamide-formamide gel of HBV-ε seg1 (lane 1) extended by a single m^6^A segment (lane 2) and then a 9 nt segment (lane 3) by SegModTeX. **Right:** Overlay of 2D-NOESY NMR spectra of the m^6^A modified HBV-ε (orange) and ordinary T7 transcribed unmodified HBV-ε RNA (black). The asterisk denotes the cross-peak of the N^6^-methyl protons and the H8 proton of residue G25 **(c) Left:** Schematic and polyacrylamide-formamide gel of tRNA^lys3^ RNA seg1 (lane 1) extended by a 12 nt seg2 containing Ψ (lane 2) followed by a 44 nt seg3 (lane 4) using SegModTeX. **Right:** Overlay of 2D-NOESY NMR spectra of the Ψ modified tRNA^lys3^ (orange) and T7 transcribed AC protonated (black) and GU protonated (green) tRNA^lys3^. The asterisk denotes the H5 proton of U27 in the non-modified tRNA^lys3^ at position 27 which is not present in the Ψ modified tRNA^lys3^ while other peaks are unaffected. Seg1 input equivalent denoted in brackets for SegModTeX products. For larger ssDNA templates full equivalents were not loaded due to high dilution volumes in the DNase treatment. Lack of input segment in product lanes shows complete product turnover.

As for the specification of 5′ ends, one current limitation when making any RNA construct, or in this case, preparation of seg1, is that T7 polymerase only initiates transcription from at least one guanosine with T7 class III promoter or with lower efficiency adenosine with a class II ϕ2.5 promoter^29,30^. Since SegModTeX extension of RNA is a novel function of a mutant DNA polymerase, we surmised it could also add RNA onto DNA segments, without sequence constraints. Hence, we tested the extension of a short DNA segment annealed onto a longer ssDNA template encoding HBV-ε. The RNA sample obtained after DNase digestion is identical to that made by T7 polymerase (Fig. 5a). Therefore, SegModTeX can be used to synthesize the first RNA segment as well, making its design independent of any sequence constraints.

### Site-specific incorporation of modified NTPs by SegModTeX

Many RNAs are modified in cells to control function. The most prevalent modifications are N^6^-methyladenosine (m^6^A), N^1^-methyladenosine (m^1^A), inosine (I), 2′-O-methylation (2′-O-m), 5-hydroxymethylcytosine (hm^5^C), 5-methylcytosine (m^5^C), and pseudouridine (Ψ)^31^. Cozen et al. showed that the latter two could substitute for Cs and Us^19^. Given that SegModTeX can be used for multi-segment extension, we wanted to test if we can site-specifically introduce these and other biologically relevant modifications.

We first tested incorporation of Ψ at position 27 in tRNA^lys3^ and m^6^A at position 26 (HBV-genome: 1907) in the distal HBV-ε stem-loop^32,33^. Both Ψ and m^6^A are widely used modifications and occur naturally in these constructs. In the first step, we used the same template:seg1 setup as before (Fig. 2), except that the single base extension in HBV-ε was carried out in the presence of m^6^ATP and then extended to completion in the second step (Fig 5b). Similarly, the 12 nt seg2 extension for tRNA^lys3^ was carried out in the presence of ΨTP, where U is specified only once in the sequence. A subsequent extension with regular rNTPs was used to complete the tRNA^lys3^ to 76nt (Fig. 5c). NMR assignments confirmed incorporation of the m^6^A at the correct position 26 in HBV-ε through the presence of an upfield shifted resonance (~ 2.8ppm)^3^, which is absent in the sample made by T7 polymerase without an m^6^A modification (Fig. 5b). Similarly, NMR assignments of tRNA^lys3^, confirmed the incorporation Ψ at position 27 by the expected disappearance of the H5 resonance of U27, which is seen in the samples made by T7 polymerase (Fig. 5c). In both cases, no other resonance of m^6^A or Ψ were observed and the spectra showed that the RNAs folded similar to their unmodified counterparts, indicating that the incorporation occurs accurately and only at the specified site.

**Figure 6:**
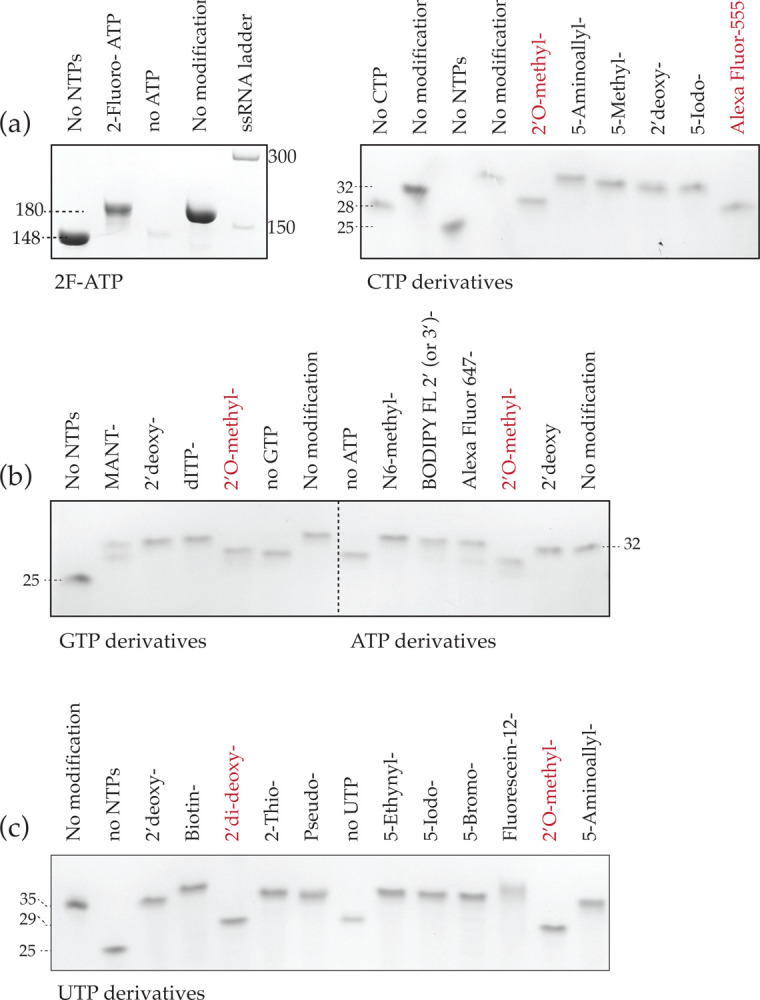
Site-specific incorporation of modified NTPs by SegModTeX Extension of 25 nt HBV-ε seg1 using a set of three regular rNTPs and a fourth modified NTP **(a) Right:** Extension of a 148nt RNA seg1 (lane 1) with CTP, UTP, GTP, and 2-^19^F-2-^13^C-ATP by a 30 nt segment encoding 5 As (lane 2) to the expected length using regular rNTPs only (lane 4). Lack of ATP analogues stalls reaction early on (lane 3). **Left:** CTP analogues incorporation in a 7 nt extension reaction are incorporated as efficiently as regular rNTPs (lanes 2 and 4) whereas no CTP, 2′O-modified (red), and Alexa Fluor-555-aha NTPs (red) stall the reaction. **(b)** As in (a) lack of GTP and ATP analogues and 2′-O-modified NTPs (red) stall the reaction, whereas all others are extended like the regular rNTP control (lanes 7 and 14) **(c)** UTP analogue incorporation in a 10 nt extension reaction showing the stalled reaction without any UTP analogue (lane 2), 2′di-deoxy-UTP (lane5, red) or a 2-’O-modification (lane 13, red) whereas all other analogues are incorporated like regular rNTPs (lane 13).

Finally, to further advance the NMR field to tackle large RNAs (>70 nt), we also introduced 2-^19^F-2-^13^C-ATP, which is of special interest to NMR spectroscopists, because of the very beneficial TROSY properties of this spin pair. As expected, 2-^19^F-2-^13^C-ATP was readily incorporated by extension of seg2 of the 7SK snRNA_148/178_ (Fig. 6a, left). We next tested other biologically relevant NTPs, including inosine, 5-methyl-C, 2-thio-U, and 2′O-methyl-nucleotides. Similarly, we also probed incorporation of NTP analogues that can be used for various biochemical techniques: i) Alexa-Fluor-647-ATP, BODIPY FL ATP 2′ (or 3’), Alexa-Fluor-555-aha-dCTP, MANT-GTP (2′-(or-3’)-O-(N-Methylanthraniloyl)), and Fluorescein-12-dUTP for fluorescence labeling; ii) 5-Aminoallyl-CTP, 5-Aminoallyl-UTP, and 5-Ethynyl-UTP for crosslinking; iii) biotin-16-UTP for affinity biology; iv) 5-Bromo-UTP, 5-Iodo-UTP, and 5-Iodo-CTP for halogeno labeling; and v) dNTPs and ddNTPs for chain termination studies.

To test these analogues, we made various HBV-ε templates that encoded each modified base only at a single specified site (position 29 of 35 for modified UTPs and position 28 of 32 for all other modified NTPs). Extension stalls at expected lengths in the absence of the specified NTPs, whereas it goes to completion with most modified NTPs: The HBV-ε (25_seg1_) extends by 7 nucleotides (for CTP- (Fig. 6a, right), GTP-, and ATP-analogues (Fig. 6b)) or 10 nucleotides (for UTP-analogues, Fig. 6c). As expected, ddNTPs cause chain termination at the expected site after incorporation. Similarly, 2′-O-methyl-NTPs and Alexa-Fluor-555-aha-dCTP are incorporated but unfortunately also cause similar chain termination. All other modified NTPs were incorporated very efficiently, with the exception of MANT-GTP and Alexa-Fluor-647-ATP with incomplete extensions of approximately 50% and 75%, respectively. In summary, this method allows for incorporation of a wide range of nucleotide analogues in RNA at specific sites with relative ease.

## Discussion

RNA biology is a rapidly advancing field; however, the construction of RNA samples with native modifications remains challenging. Furthermore, NMR, one of the main methods for structural analysis accounting for 35% of deposited RNA structures^17^, is severely limited due to the complexity of assigning spectra without the ability to isotopically label individual segments. Finally, biochemical methods such as FRET, RNA-crosslinking, etc., are confined to end-labeling or cumbersome ligation techniques to achieve site-specific incorporation. In this paper we showcase the use of a mutant *Tgo* DNA polymerase for ‘Segmental labeling and site-specific Modifications by Template-directed eXtension’ (SegModTeX) — a one-stop protocol to address all these limitations.

First, unlike ligations, the extension reaction goes to completion, which addresses the biggest hurdle of segmental labeling. Thus, insofar as the product is recovered efficiently, the yield can approach 100%. This in principle allows for multiple rounds of extensions. Second, although the enzyme has been mutated to accommodate rNTPs, it has not resulted in a loss of complementary base recognition, maintaining the high fidelity of polymerases. This directly benefits junction homogeneity because mismatched 3′-ends are consequently selected against for extension; therefore, the end product only contains the desired sequences. Similarly, unlike RNA polymerases, no RNA self-templating occurs^34^, leading to homogeneous 3′-ends to the extent that the ssDNA template is accurate. In fact, use of asymmetric PCR to make high quality ssDNA template renders the process independent of any sequence length constraints that arise from restrictions of chemical synthesis. Third, since all extensions require the same optimized conditions, no further optimization for reaction conditions or special sequence considerations are required. Moreover, compared to even a single-step ligation protocol, which involves separate preparation of the two segments that are appropriately protected or primed for ligation followed by splinting and addition of ligase, SegModTeX allows for a one-step reaction to achieve segment joining. Fourth compared to T7 polymerase-driven RNA production, this method has an additional advantage that no base-type restrictions exist for the initiating nucleotide. Furthermore, the retention of DNA-primed extension by the enzyme allows seg1 to have no minimum length requirement because the RNA segment alone does not have to be long enough to anneal. Finally, the salient feature of SegModTeX is the ability to use multi-step extensions to site-specifically incorporate a plethora of modified NTPs at any desired position(s) without sequence constraints around the modified site(s). For example, one could specifically modify or label any cytosine, even if it is flanked by other cytosines, via single nucleotide extensions.

This versatility and ease of SegModTeX has the potential to transform RNA structure determination by NMR, as structures of smaller domains can be solved in its larger context due to the drastic reduction of spectral crowding. This, along with the current progress in assignment prediction software can allow for a rapid workflow for characterizing large RNAs currently outside the scope of solution NMR^35^. In addition, this technique can be used to build more sophisticated tools for existing methods. For example, currently RNA FRET is mostly limited to labels at the two ends, whereas SegModTeX permits easy incorporation of fluorescent labels at any position on an RNA, thus granting access to the whole molecule. We see similar potential for selective halogeno labeling for x-ray crystallography, crosslinking, or affinity tags.

## Methods:

### TGK polymerase:

The plasmid for TGK-polymerase expression was cloned to encode an N-terminal Glutathione-S-transferase, a PreScission protease cleavage site, and the polymerase insert between the BamHI and NotI restriction sites of a pGEX-6P-3 vector. Plasmid DNA was transformed into One Shot^™^ BL21 Star^™^ cells and grown to 2L from overnight cultures. Subsequent IPTG induction at ~O.D. 0.7 was followed by 3–4 h growth. Cells were concentrated by centrifugation, resuspended in 40 ml lysis buffer (25 mM Tris pH 7.5, 300 mM NaCl, 1 mM EDTA, 2 mM DTT), and sonicated on ice with six 1-min cycles at 60%−70% duty, with 1 min cool down between each pulse. Lysate was incubated with washed Pierce^™^ Glutathione Agarose slurry from ThermoFisher (cat. #16101) in lysis buffer for 2 h at 4 °C. Subsequently, the beads were washed with 750 ml lysis buffer and cleaved overnight in 10 ml of elution and storage buffer (0.01 M Tris-HCl pH 7.6, 0.1 M KCl, 0.1 mM EDTA, 1 mM DTT, 0.1 % v/v Triton X-100, 5 % glycerol) with 60 μl aliquot of PreScission protease (13 mg/ml made inhouse). After elution from the column, TGK was stored at 4 °C for short-term or diluted with one volume glycerol at −20 °C for long-term.

### DNA template design:

The ssDNA templates contain a 3′end reverse complementary to the 3′ end of the RNA segment for their hybridization. The length should translate to a melting temperature (T_m_) of ~65 °C, though lower melting temperatures are possible if the reaction is run at lower temperatures (see below). Longer hybridization regions do not affect SegModTeX. As the template is single-stranded, it is advised to avoid long self-complementarity at the 3′ends to avoid the template serving as primer for the TGK polymerase in an off-target reaction, however, as the reaction occurs at 72 °C, we have not encountered this problem in any tested construct thus far. Moreover, if self-templating is observed non-extendable ssDNAs with di-deoxy or 3′-monophosphate ends can be used.

### DNA template preparation:

For SegModTeX templates shorter than 100 nt, ssDNA was ordered from Integrated DNA Technologies (IDT) and if necessary, purified on a 6% polyacrylamide 25% formamide sequencing gel. SegModTeX templates longer than 100 nt were prepared using asymmetric PCR as described in^28^, extracted via a 2-step ammonium acetate (2.5 M) and 70% ethanol precipitation, and subsequently washed and concentrated with H_2_O in Amicon^®^ Ultra-15 Centrifugal Filter Units.

Similarly, for control samples made by T7 polymerase, shorter templates were ordered from IDT, with 2′-O-methylated 5′-ends^36^. For the longer 7SK snRNA samples, plasmids containing the T7 promoter, insert, and SmaI recognition sequence were cloned in between the EcoRI and BamHI restriction sites of a PUC19 vector. Plasmid DNA was prepared for PCR amplification from a 2mL overnight culture of NEB 5α Competent *E. coli* (C29871) transformed with the plasmid. Subsequently, the precise template sequences were amplified using primers ordered from IDT and EconoTaq^®^ PLUS 2X Master Mix. PCR products were extracted via a 2-step ammonium acetate (2.5M) and 70% ethanol precipitation.

### RNA seg1 preparation:

RNA seg1 for the various constructs were synthesized by *in vitro* transcription using T7 RNA polymerase. Chemically synthesized RNAs were also tested for constructs with RNA seg1 smaller than 26 nt (ordered from IDT). Resuspended RNA segments from *in vitro* transcription and ethanol precipitation or chemical synthesis were directly used for SegModTeX. For most reactions, the RNA was PAGE purified before every round of SegModTeX to avoid any carry-over of rNTPs from previous reactions which SegModTeX might mistakably incorporate into the new segment as well. However, we found that for smaller RNA constructs (<70nt) ammonium acetate – ethanol precipitation successfully removes rNTPs from the previous step.

### SegModTeX reaction conditions:

#### Reaction mix:

All SegModTeX reactions were conducted according to the following optimized protocol: 1x ThermoPol buffer (NEB), 15 mM MgSO4, 1.5 mM rNTPs, 0.1 mM RNA seg:ssDNA template, and TGK polymerase to a final concentration of ~0.1 mg/ml were mixed at 4 °C. For large RNAs, SUPERase•In^™^ RNase Inhibitor was added according to the manufacturer’s specifications.

#### NTPs:

The NTP mix contained the individual bases at concentrations according to their demand in the extension reaction. For rare or expensive NTPs, ratios as low as 15-fold excess per base incorporation were successfully tested. Higher concentrations of NTPs require special considerations for the DNase treatment step (see below).

#### Temperature:

The reaction mixture was incubated at either 65 °C (for RNA segments with a calculated T_m_ of the template hybridization sequence of less than 63 °C) or else 72 °C. If experimental design requires very short RNA:DNA hybridization regions (< ~15nt), extension can be started at lower temperatures to allow for the addition of initial bases so as to increase the T_m_, after which the temperature can be increased to allow for fast completion. Under optimized conditions, we observed SegModTeX starting to extend RNAs even at room temperature (data not shown).

#### Time:

The SegModTeX reactions were incubated for 20 min (extensions of fewer than 30 nt) to up to 90 min. Extended incubation time of up to 90 min can be beneficial for long extensions or highly structured segments but did not impact RNA integrity in our experiments. As TGK is a thermostable polymerase, heat-denaturation, either at the beginning or in between the incubation, akin to PCR cycles, was successfully tried and can be used for reaction optimization but was not required for any RNA sample shown. Moreover, we found that increasing the polymerase concentration was an easier and effective way to achieve extension completion.

#### DNA removal and RNA purification:

Template DNA was digested with Turbo DNase according to the manufacturer protocol (37 °C, 15 min). It is important to note that incomplete DNA digestion has a detrimental impact on data quality due to the difficulty in separating it from the RNA in subsequent steps. Hence, for the digestion, special consideration should be given to the maximum allowed DNA concentration as specified in the Turbo DNase protocol, as high template and NTP concentration are inhibitory. This also limited the amount of SegModTeX product added in polyacrylamide gel wells due to volume constraints. In most cases, three-fold dilution of the reaction mix was necessary and sufficient to insure complete digestion. To stop the reaction, EDTA was added equimolar to the Mg^2+^ concentration and denatured at 95 °C for 2 min.

For purification without PAGE, ½ volumes 7.5 M ammonium acetate was added, the sample vortexed for 10 s, placed on ice for 10 min and centrifuged at 15,000 g for 20 mins, 4 °C. This step is necessary to remove the enzymes in the sample, which might interfere with applications downstream. In a new tube, the supernatant and 4 volumes ethanol were added, mixed, and precipitated for 2 h at −80 °C. The centrifuged pellet was washed twice with 80% cold ethanol, dried at room temperature for 6 h, and resuspended in H_2_O. The sample was then ready for another round of SegModTeX.

For PAGE purification, the digestion reaction was precipitated using 0.3 M sodium acetate in 4 volumes ethanol at −80 °C and subsequently resuspended in RNA gel-loading buffer (50% formamide, 25 mM EDTA) and purified using urea-PAGE with 25% formamide. The choice of purification method depends on the subsequent use of the RNA. As the extension of RNA in SegModTeX is essentially 100%, size-based separation of the RNA product is not necessary in general. However, as the sample is in a mix of salts, proteins, NTPs, dNMPs and DNA oligos, precipitation or filter-based methods might be insufficient for highly sensitive applications, such as NMR.

### NMR data acquisition and resonance assignment:

RNA samples were suspended in the appropriate buffer (MLV: 5 mM Tris•HCl, 10mM NaCl, pH: 7.0, 37 °C; 7SK: 5 mM Tris•HCl, pH: 7.0, 25 °C; HBV: D_2_O, pH: 7.0, 25 °C; tRNA: 10 mM Tris•HCl, 10 mM NaCl, 7 mM MgCl2, pH: 7.5 buffer, 37 °C). All NMR experiments were acquired in 5 mm Shigemi tubes with Bruker 700 and 800 MHz instruments containing cryogenic probes. Spectra for observing non-exchangeable protons were collected in 100% D_2_O, for exchangeable protons in 90% H_2_O.

### Sequencing:

The DNA oligo (5′TAATACGACTCACTATAGGGTCTCTTGTTCATGAGTCATGG3′) was 5′ phosphorylated with T4 PNK (NEB, #M0201S) and ligated onto the 3′ ends of PAGE purified SegModTeX and T7 constructed 7SK snRNA NMR samples with T4 RNA ligase 1 (NEB, #M0437M) according to the manufacturer’s protocols. Reverse transcription was performed with a short (25 nt) primer (5′TCCATGACTCATGAACAAGAGACCC3′) using GoScript^™^ Reverse Transcriptase (Promega A5003). PCR amplification of the RT product was done with EconoTaq^®^ PLUS (LGC Biosearch Technologies #300331) with the RT primer and primers complementary to the 5′ start of 7SK snRNA. The agarose-gel purified PCR products were sent for Sanger sequencing.

### Gel quantification:

Gel band intensities for MANT-GTP and Alexa-Fluor-647-ATP were measured by ImageJ (NIH). Intensity of top band divided by the total intensity of top and bottom band.
